# A meta-analytic review of measurement equivalence study findings of the SF-36® and SF-12® Health Surveys across electronic modes compared to paper administration

**DOI:** 10.1007/s11136-018-1851-2

**Published:** 2018-04-16

**Authors:** Michelle K. White, Stephen M. Maher, Avery A. Rizio, Jakob B. Bjorner

**Affiliations:** Optum, 1301 Atwood Avenue, Suite 311N, Johnston, RI 02919 USA

**Keywords:** SF-36, Patient-reported outcomes, Equivalence, Meta-analysis, Electronic, PRO, ePRO, Measurement

## Abstract

**Purpose:**

Patient-reported outcome (PRO) measures originally developed for paper administration are increasingly being administered electronically in clinical trials and other health research studies. Three published meta-analyses of measurement equivalence among paper and electronic modes aggregated findings across hundreds of PROs, but there has not been a similar meta-analysis that addresses a single PRO, partly because there are not enough published measurement equivalence studies using the same PRO. Because the SF-36^(R)^ Health Survey (SF-36) is a widely used PRO, the aim of this study was to conduct a meta-analysis of measurement equivalence studies of this survey.

**Methods:**

A literature search of several medical databases used search terms for variations of “SF-36” or “SF-12” and “equivalence” in the title or abstract of English language publications. The eight scale scores and two summary measures of the SF-36 and SF-12 were transformed to norm-based scores (NBS) using developer guidelines. A threshold of within ± 2 NBS points was set as the margin of equivalence. Comprehensive meta-analysis software was used.

**Results:**

Twenty-five studies were included in the meta-analysis. Results indicated that mean differences across domains and summary scores ranged from 0.01 to 0.39 while estimates of agreement ranged from 0.76 to 0.91, all well within the equivalence threshold. Moderator analyses showed that time between administration, survey language, and type of electronic device did not influence equivalence.

**Conclusions:**

The results of the meta-analysis support equivalence of paper-based and electronic versions of the SF-36 and SF-12 across a variety of disease populations, countries, and electronic modes.

## Introduction

As the importance of patients’ perspectives on their health and wellbeing is increasingly recognized in healthcare evaluation, particularly in treatment efficacy studies [1], the use of patient-reported outcome (PRO) measures has increased, as has the demand for rigorous research providing evidence of the reliability and validity of PRO measures for their intended use [1–3]. Many PRO measures were originally developed for paper administration, but have been subsequently altered to allow for electronic administration, for example, via computer, tablet, or handheld device [2]. Reflecting the increased availability and demand for electronic PRO measures, approximately 45% of pharmaceutical companies had adopted their use in some clinical trials for drug development as of 2014 [4].

While the electronic administration of PRO measures has advantages for both researchers and study participants, including management of skip patterns and reductions in missing data [2, 3, 5, 6], it cannot be assumed that data collected using a different mode of administration will produce equivalent scores to that of the original mode [2, 7]. The ISPOR ePRO Good Research Practices Task Force advised which types of modifications made to PRO measures should be considered minor, moderate, or substantial, depending on the ways in which these modifications could affect participants’ responses to survey items [2, 3]. The guidelines also advised on the type of evidence required to support mode equivalence for each level of modification [2, 8]. Minor changes that do not include substantive modifications to instructions or item content are unlikely to greatly influence participant responses, so the level of evidence recommended to support mode equivalence is usability testing and cognitive debriefing interviews [2]. Moderate modifications (e.g., changes in item wording or significant changes in presentation) may have unintended consequences on participant responses, and thus both equivalence testing and usability testing are recommended [2]. Substantial changes include changes to item or response choice wording, and in these cases a psychometric evaluation study is recommended.

Many individual studies have been conducted to assess the measurement equivalence of paper and electronic-based modes of PRO administration, with three subsequent meta-analyses aggregating these findings [6–8]. The first, including studies published before 2007, reported that the absolute mean scale-standardized difference between modes was 2% of the scale range, while the pooled correlation between paper and computer-based administrations was 0.90 [7]. The second, including studies published between 2007 and 2015, reported comparable results, as the absolute mean scale-standardized difference between modes was 1.8% of the scale range, and the pooled correlation between modes was 0.88 [8]. Taking a different meta-analytic approach, and including papers published between 2007 and 2014, a third study reported that 78% of the papers reviewed reported equivalence between paper and electronic PRO measures [6]. All three meta-analyses concluded that scores derived from paper and electronic PRO measures are equivalent when there has been a quality migration from paper to electronic mode (i.e., a migration that includes only the changes necessary to increase usability of the survey in the new format, with minimal changes to survey content), regardless of type of electronic mode (computer, tablet, or handheld device).

While the conclusions of these three meta-analyses could be interpreted as obviating the need to conduct further mode equivalence studies, it should be noted that the three studies included findings from more than 100 different PRO measures covering many different response formats and constructs. It is plausible that mode effects may particularly pertain to specific item types or constructs; such specific effects may have been attenuated in the global evaluation of mode effects used in previous meta-analyses. Although the three meta-analyses found that differences between scores derived from paper and electronic-based PRO measures are small, factors that influence agreement between scores were identified. For example, greater agreement was observed between paper and tablet administrations than between paper and an older technology, personal digital assistant (PDA), administrations [8]. Agreement also varied by other factors, including the time interval between administrations and average age of participants [8]. No meta-analysis to date has investigated the equivalence of a single PRO measure. Rather, they collapsed across PRO type, basing conclusions on a combination of item types and constructs.

The goal of this study is to examine the measurement equivalence of a single, multi-scale, generic PRO assessment that is designed for use across a variety of populations, applying established guidelines [2, 3, 9] to investigate the measurement equivalence of paper and electronic modes of the SF-36 and SF-12 Health Surveys (SF-36, SF-12). The SF-36 was identified as the most frequently used PRO measure in studies of mode equivalence [6], and is also the most widely used generic PRO measure in clinical trials [10]. The SF-36 and SF-12 were originally developed for paper administration, but have been modified for electronic administration. Specifically, single-item formats of the SF-36 and SF-12 were developed for administration on smaller screens. The percentage of SF-36 and SF-12 surveys that were licensed for electronic use increased from 11.3% of all commercial licenses in 2011 to 41.7% in 2016, underscoring the need to more fully explore the measurement equivalence of electronic formats (licensing statistics provided through personal communication with licenser, Jan 2017). As such, a meta-analytic approach provides the best opportunity to comprehensively evaluate and synthesize the available data.

## Methods

### Measures

The SF-36 is a 36-item self-report survey that assess eight domains of functional health and wellbeing: physical functioning (PF), role limitations due to physical problems (role-physical, RP), bodily pain (BP), general health perceptions (GH), vitality (VT), social functioning (SF), role limitations due to emotional problems (role-emotional, RE), and mental health (MH). Scores can be calculated for each of the eight domains of the SF-36. In addition, two summary scores [physical component summary (PCS) and mental component summary (MCS)] can be calculated from the eight scales [10]. The first version of the SF-36 was developed in the 1980s. The second version (SF-36v2) was subsequently developed as a revision to the first survey, incorporating changes based on additional testing, cross-cultural validation, developing norm-based scoring, and the implementation of missing data estimation algorithms. The SF-12 and SF-12v2 assess the same eight domains as the SF-36, using 12 items of the SF-36 and SF-36v2, respectively. The SF-12 (and SF-12v2) allows scoring of the PCS and the MCS, which have been found to strongly agree with the SF-36-based PCS and MCS scores [11].

Single-item formats of both surveys have been developed for (1) tablets (typically seven inch diameter screen size or larger) and (2) handheld devices (e.g., smartphone or PDA, typically smaller than seven-inch-diameter screen size). In addition to presenting only one item per screen, other minor changes were implemented in the migration to single-item format, including displaying response choices vertically instead of horizontally to better accommodate smaller screens, and changing the instruction to read as “Select the one response” instead of “Mark an X in the one box.” [12].

### Literature search and screening

**Fig. 1:**
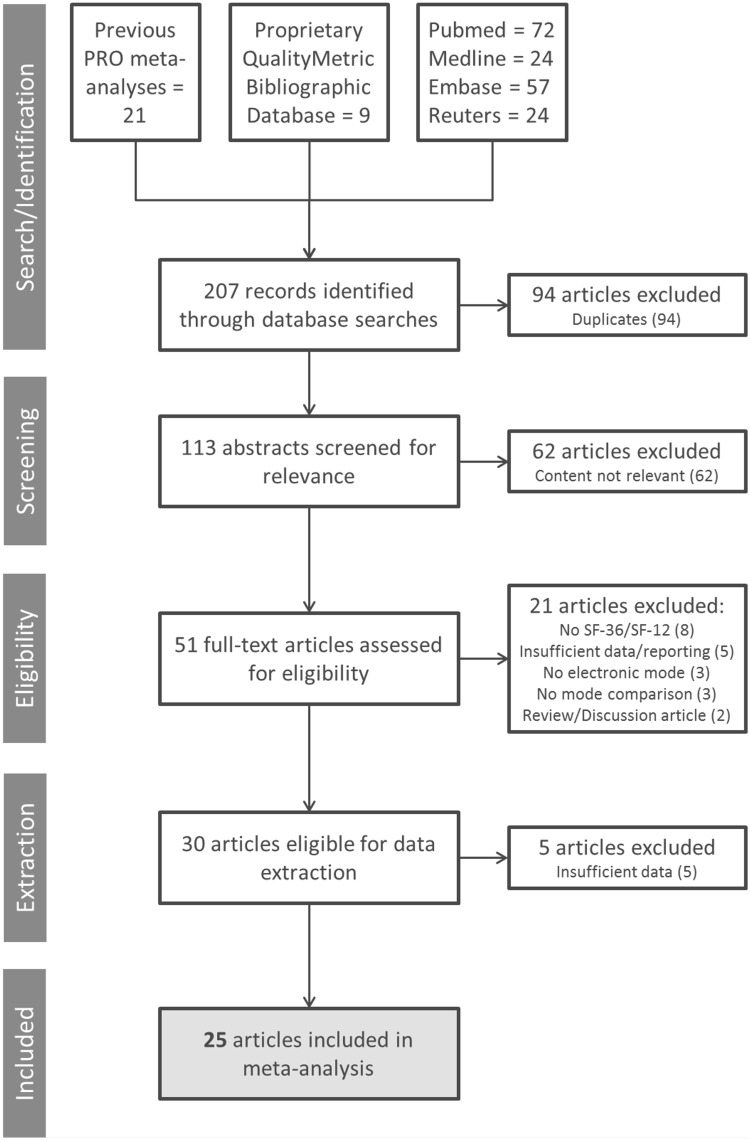
Article selection. Multiple databases were searched for articles that assessed the measurement equivalence of SF-36 or SF-12 Health Surveys. Articles were screened according to established inclusion/exclusion criteria, which resulted in the inclusion of 25 articles in the meta-analysis

A comprehensive literature search was conducted to identify published manuscripts that examined measurement equivalence between paper and electronic versions of the SF-36 and SF-12 (see Fig. 1 for diagram of selection process). First, databases of medical literature, including PubMed, Embase, Medline, and Reuters were searched for peer-reviewed articles, conference proceedings, or published abstracts that included titles or abstracts that fit the following search string: [SF-36 or SF-12] and [Internet or touchscreen or web or tablet or computer or electronic] and [paper] and [compar* or equiv*] and [questionnaire]. Asterisks were used to capture multiple words that have the same stem (e.g., equivalent, equivalence), and several variants of the terms SF-36 and SF-12 were used to find articles that used different naming conventions for the survey (e.g., SF36, short form 36, SF12, short form 12). The searches were conducted in October 2016 and were not limited by year of publication. Second, the same terms were used to search a bibliographic database maintained by Optum that houses more than 29,000 publications that report using various SF Health Surveys. Third, the three previous meta-analyses were also screened for publications that used the SF-36 or SF-12 to ensure no studies were missed by the first two searches. This combination of search strategies identified 113 unique publications. The reference sections of these 113 publications were also screened to further ensure that no relevant publications were missed, though this did not result in inclusion of any additional articles. To be included in the meta-analysis, studies had to be published in English, contain a sample of adults (≥ 18), use a paper to electronic migration of either the SF-36 or SF-12 (v1 or v2), and test measurement equivalence using one of two indices: (1) mean differences; (2) agreement [i.e., intraclass correlation coefficient (ICC), Pearson product-moment correlation, Spearman rho, weighted kappa]. Studies examining only paper to interactive voice response (IVR) migration were excluded. Sixty-two studies were excluded for not meeting these criteria after abstract review, with an additional 21 articles excluded after full-text review. Five additional articles did not include sufficient statistical information and were consequently excluded during data extraction, resulting in a total of 25 articles for inclusion in the meta-analysis.

### Analysis methods

Of the 25 studies that met the inclusion criteria discussed above, 15 studies reported differences in means, and 20 studies assessed agreement between administration modes using measures of statistical association such as the intraclass correlation. Separate meta-analyses were performed for analyses of differences between means and analyses of agreement statistics. Ten studies reported mean differences and analyses of agreement and thus were included in both analyses. As different studies analyzed different subsets of domains or summary scores, the number of studies for any given domain or summary score varied across the two analyses (Table 1).

**Table 1:** Number of studies included in the meta-analysis that reported mean differences or agreement for each SF domain and component summary score

	Physical functioning	Role-physical	Bodily pain	General health	Vitality	Social functioning	Role-emotional	Mental health	Physical component summary	Mental component summary
Mean Difference	11	10	11	11	11	10	11	11	9	9
Agreement	18	17	18	18	18	17	17	18	16	16

In total, 25 studies were included in the meta-analysis. Some studies reported both mean differences and agreement, while others reported only one or the other

For the analysis of studies reporting differences between paper and electronic versions, we used the SF-36 and SF-12 developer scoring software and related guidelines to calculate scores and convert to norm-based scores (NBS) (mean = 50, *SD* = 10) based on a US general population normative sample, with higher scores indicating better health-related quality of life (HRQoL). SF-36 and SF36v2 (and SF-12/SF-12v2) were scored according to the appropriate normative sample for that survey. Since differences in NBS are roughly comparable across versions, no distinction between versions was made in the subsequent analyses. For the 15 studies in the mean difference meta-analysis calculation, effect size (ES) estimates were derived for four studies from paper and electronic mean scores, standard deviations, and correlations between modes; three studies provided mean differences, standard deviation of the mean difference, and correlation between modes; three studies provided mean differences and within-subject *t* values; two provided mean differences and within-subject *t* and *p* values. Along with sample size, these statistics allowed for estimation of the pooled standard deviation, making a point estimate and confidence interval (CI) across studies possible. Three studies provided mean scores for the two modes along with the correlation between the modes, but no measure of dispersion. In these cases, a standard deviation of ten (equal to the general population standard deviation) was assigned for both paper and electronic versions. Mode equivalence studies typically use the minimally important differences (MIDs) provided by the developer as the margin of equivalence threshold. For the SF-36, MIDs vary by scale and range from two to four points [13]. To simplify interpretation and not have a different equivalence threshold for each scale, we chose to use the most conservative MID (two points) for all scales. As such, we specified that the 95% CI for each scale should be within a margin of equivalence set at ± 2 points.

For studies investigating agreement between paper and electronic administration, measures of agreement varied by study, and included the ICC, Pearson product-moment correlation (Pearson’s *r*), and Spearman’s rank correlation coefficient (Spearman’s rho). If multiple measures of agreement were reported the ICC was preferred, in accordance with the ISPOR ePRO Good Research Practices Task Force recommendations, followed by Pearson’s *r*, and then Spearman’s rho [2]. The meta-analysis of correlation coefficients included ICC from 13 studies, Pearson’s *r* from seven studies and Spearman’s rho from one. All three coefficients range from − 1 to 1, where higher positive values indicate a higher degree of agreement. Consistent with Gwaltney et al.’s [7] meta-analysis of mode equivalence, our meta-analysis combined different coefficient types together in the same analysis, which were converted to Fisher’s *Z*. As a correlation coefficient is itself an ES, a meta-analysis synthesizing correlations can be conducted using these coefficients and each study’s sample size to produce a point estimate across studies that reflects the degree of agreement between modes of administration. As criterion for equivalence, we specified that the 95% CI for each scale should be above 0.7, which is a more conservative approach than that recommended by ISPOR ePRO Good Research Practices Task Force [2].

For both analyses, Comprehensive Meta-Analysis Version 3 software was used to aggregate and synthesize studies for each domain and for summary scores separately. Studies were inversely weighted by standard error so that studies with larger sample sizes were given greater weight. A random effects model was used to calculate the pooled ES estimates.

Meta-analytic tests of moderators were performed in the same way as the main analyses specified above, but included respective moderators as between-studies factors to test for differences between moderator-defined studies. Tests of moderators were performed for variables that may reasonably affect mode equivalence if they were available in sufficient numbers per group, defined as more than two studies, considering that a random effects model was used [14, 15]. Both difference and correlation analyses included lag time (categorized into ≥ 24 or < 24 h lag time between administrations) and language of administration (English or other) as moderators. For the correlation analysis only, a sufficient number of studies were available to compare type of electronic mode [computer (desktop or laptop) vs. either tablets or smaller handheld devices].

## Results

### Overview of articles

Of the 25 articles incorporated into the meta-analysis, 20 used the SF-36, and five used the SF-12 [16–40]. As shown in Table 2, the selected articles assess measurement equivalence using a range of electronic modes and participants from multiple groups/disease conditions. Twelve studies reported administering the SF-36/12 on a personal computer (PC), six on a tablet, four on the web, two on a PDA, and one on a handheld device. Participants in these studies were recruited from a variety of clinical areas, including cardiology, rheumatology, and psychiatry, and the majority of studies (56%) reported that the survey was administered in English.

**Table 2:** Characteristics of studies included in meta-analysis

Study description										Equivalence indices		
										Mean difference	Correlation	
Author	Year	eMode	Design	Form	*N*	Population	Mean age (SD)	Time lag	Survey language		ICC	PPM
Basnov et al.	2009	Web	C/R	SF-36	41	Gynecology	47.2 (9)	14.7 days	Danish		●	
Bliven et al.	2001	PC	C/R	SF-36	55	Cardiology	51.9	Same visit	English	●	●	
Broering et al.	2014	Web	C/R	SF-36	209	Prostate cancer		2–5 days	English	●		
Burke et al.	1995	PC	C/R	SF-36	138	Psychiatry	46	Sequential	English		●	
Caro et al.	2001	Tablet	C	SF-36	68	Asthma	48	2 h	Quebec French		●	
Chen et al.	2007	PC	C/R	SF-36	150	Patients and students	30.8	10 min	Chinese	●		
Cunha-Miranda et al.	2015	PC	C	SF-36	120	Rheumatology	50.8 (11.9)	15 min	Portuguese	●	●	
Elash et al.	2015	Tablet	C	SF-36	53	Psoriatic arthritis	55.6	45 min	English			●
Farr et al.	2013	PC	C	SF-36	102	Orthopedics		Hours–days	English			●
Frennered et al.	2010	Web	C	SF-36	79	Spine problems	54/53	1–3 weeks	Swedish	●	●	
Gudbergsen et al.	2011	Tablet	C/R	SF-36	20	Osteoarthritis	67 (7)	5 min	Danish	●	●	
Kongsved et al.	2007	PC	P/R	SF-36	553	Gynecology		Not controlled	Danish	●		
Khurana et al.	2015	Handheld	C/R	SF-36	408	Chronic disease	55	Same visit	English		●	
Kvien et al.	2005	PDA	C/R	SF-36	30	Rheumatology	61.6	5–7 days		●		●
MacKenzie et al.	2011	PC	C/R	SF-12	67	Psoriatic arthritis	53	1 h	English	●	●	
Marsh et al.	2014	Web	C/R	SF-12	53	Arthroplasty	69	1 week	English	●	●	
Naus et al.	2009	PC	C/R	SF-36	76	Female undergraduate	24.01 (8.36)	10–21 days	English	●		●
Ribeiro et al.	2010	PC	C	SF-36	50	Immune diseases	45.2 (15.3)	Same visit	Portuguese			●
Richter et al.	2008	Tablet	C/R	SF-12	153	Rheumatology	45.7 (14.4)	Same visit	German	●		●
Ryan et al.	2002	PC	C/R	SF-36	115	Various	74, 40, 16, 46	5 min	English	●		
Shervin et al.^a^	2011	Tablet/web	C	SF-36	66	Osteoarthritis	63	Not controlled	English			
Tiplady et al.	2010	PDA	C/R	SF-12	43	Rheumatology	57	45 min	English		●	
Waehrens et al.	2015	Tablet	C	SF-36	20	Fibromyalgia	47.8 (11)	5 min	Danish	●	●	
Whitehead et al.	2011	PC	P/R	SF-12	1034	Mental health	24.07(8.5)	Not applicable	English	●		
Wilson et al.	2002	PC	C	SF-36	80	Rheumatology	50 (14.7)/43 (12)	Same visit	English			●

Empty cells indicate that data were not reported for that characteristic

*C* crossover, *ICC* intraclass correlation, *P* parallel, *PPM* Pearson product moment, *PC* personal computer, *R* randomized, *SD* standard deviation

^a^Used Spearman’s Rho as measure of agreement

### Evaluating mean differences

**Fig. 2:**
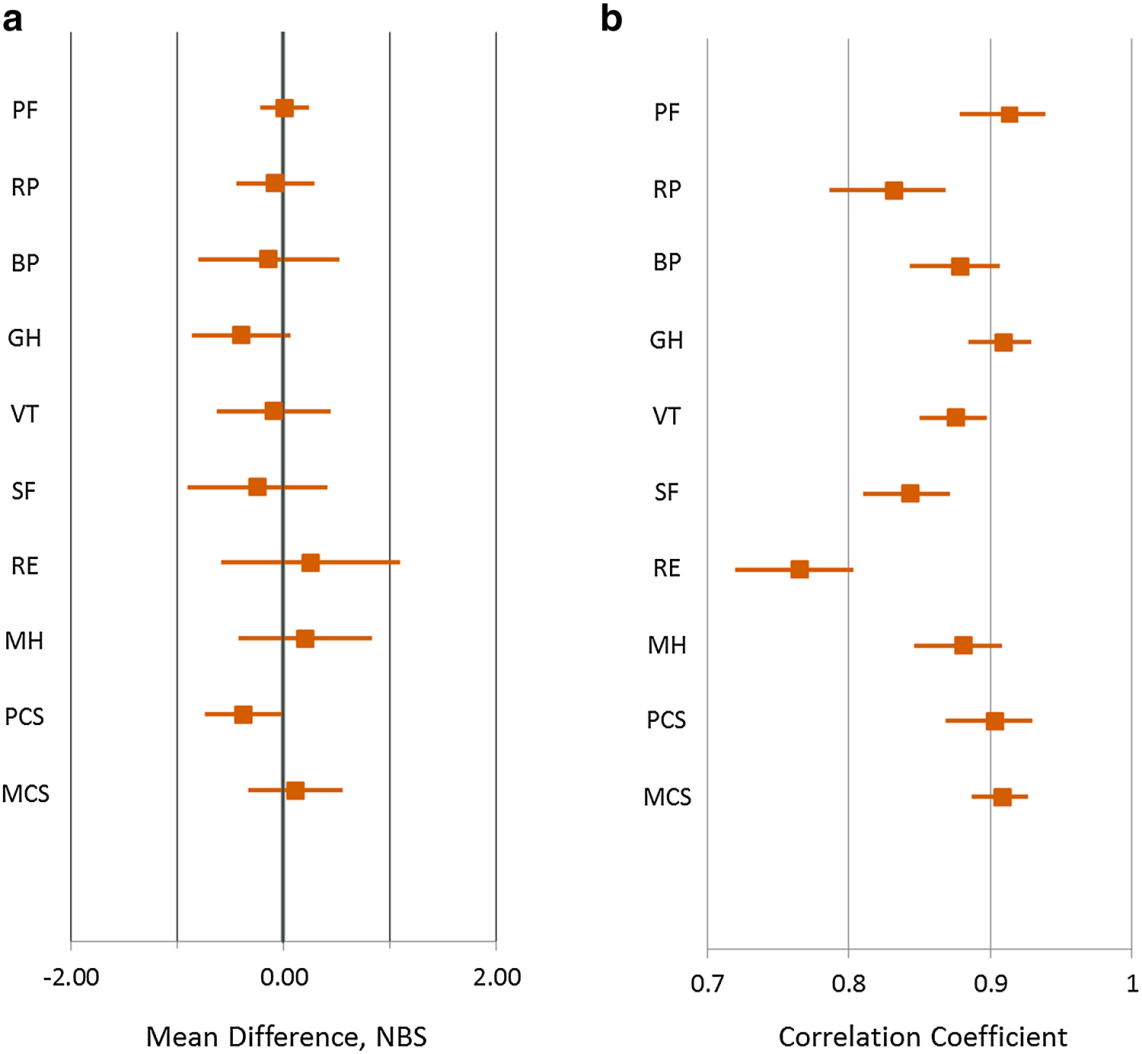
Meta-analysis of mode equivalence by SF-36 domain, as demonstrated by **a** mean differences and **b** agreement. *NBS* norm-based score

Estimates of mean differences across domains and summary scores ranged (in absolute values) from 0.01 (PF) to 0.39 (GH) (Fig. 2a). All 95% CIs were within the specified ± 2 point margin of equivalence, and except for the RE scale (95% CI − 0.59, 1.1) all CIs were within ± 1 point.

### Analysis of agreement

The results of the meta-analysis of agreement for each domain and summary score are shown in a forest plot (Fig. 2b), with point estimates and CIs for each of the domains and summary scores. The estimates ranged from 0.76 (RE) to 0.91 (PF), though the next lowest after RE was 0.83 (RP). For all scales, the 95% CI was above our specified criterion of 0.7. The summary scores, PCS and MCS, each had estimated correlation coefficient value of ~ 0.90.

### Analysis of moderators

Moderator analyses did not show significant differences for either the mean difference analyses or the agreement analyses.

Specifically, for the mean difference analyses, studies with a lag time < 24 h showed an average absolute difference across domains and summary scores of 0.29 NBS points, while studies with a lag time ≥ 24 h showed a 0.42 NBS point difference. In studies using the SF-36/12 in the English language, the average absolute difference across domains and summary scores was 0.42 NBS points, while studies using the SF-36/12 in other languages showed a 0.38 NBS point difference. For studies using desktop or laptop computers, the absolute difference across domains and summary scores was 0.01 NBS points, while the difference for smaller-screen devices (tablets/handheld) was 0.10 NBS points.

For the correlation analyses, studies with a lag time < 24 h showed an average correlation of 0.88 across domains and summary scores, while studies with a lag time ≥ 24 h showed an average correlation of 0.86. In studies using the SF-36/12 in the English language, the average correlation across domains and summary scores was 0.85, while studies using the SF-36/12 in other languages showed a correlation of 0.89. In studies using desktop or laptop computers, the average correlation across domains and summary scores for computers was 0.87, while studies using tablets/handheld showed a correlation of 0.87. However, none of these differences were statistically significant.

## Discussion

This study used a meta-analytic approach to explore the measurement equivalence of paper and electronic versions of the SF-36 and SF-12, two widely used assessments of HRQoL. Analyses of mean differences and measures of agreement support the finding that a migration from paper to electronic mode has no effect on the scores obtained from any of the SF-36 and SF-12 domain and summary scores. More specifically, the overall absolute mean difference between modes ranged from 0.01 to 0.39 points and for all scales and summary scores the 95% CI was well within pre-specified, conservative threshold of ± 2 point margin of equivalence. Scores obtained from paper and electronic modes showed notable agreement, as correlations between the modes were all significant, and ranged from 0.76 to 0.91 for each of the eight domains and two component summary scores. This agreement was found despite the highly varied populations and research purposes of the included studies. The time lag between administrations, language of survey, and screen type (size) were not shown to moderate either meta-analysis.

While no previously published meta-analysis has examined the measurement equivalence of only the SF-36 and SF-12, the observed results are similar to those from meta-analyses that collapse across several different PRO measures (Table 3) [7, 8]. Because the current study focused on a specific PRO measure (SF-36) and its shortened form (SF-12), the observed differences can be directly compared to pre-established criteria regarding MIDs, lending insight into the interpretation of the observed scores. Proposed MID values for group-level data derived from the SF-36 and SF-12 range from 2 to 4 NBS points, depending on the domain and survey (SF-36v2 versus SF-12v2) [10, 11]. The study authors used a more conservative threshold, the smallest MID of any SF-36/12 scale, of ± 2 points. It is clear from the data presented above that the mean differences between modes for each domain were well below the threshold, further verifying the measurement equivalence of various modes of administration.

**Table 3:** Comparison of relevant mode equivalence meta-analyses

	Gwaltney et al. [7]	Muehlhausen et al. [8]	Campbell et al. [6]	Current study
Review year range	Pre-2007	2007–2013	2007–2014	No restrictions; articles ranged in year of publication from 1995 to 2015
Total studies reviewed	46	72	55	25
Most common correlation coefficient used	ICC	ICC	ICC	ICC
Number of different PROs included	48	117	79	2 (SF-36 and SF-12)
Number of different electronic modalities included^a^	3: PC/laptop, tablet, PDA	4: PC, tablet/touchscreen, handheld (PDA/smartphone), IVRS	5: Internet, computer, touchscreen computer, tablet, PDA	4: Web, PC, tablet, handheld
Report mean differences	Average = 0.2% of the scale range Range = − 7.8 to 7.6%	Average = 0.037	NA	Range = 0.01–0.39
Reported agreement	Average = 0.90	Average = 0.88 Range = 0.65–0.99	NA	Range = 0.76–0.91

*ICC* intraclass correlation coefficient, *IVRS* interactive voice response system, *PC* personal computer, *PDA* personal digital assistant

^a^With the exception of the inclusion of IVRS by Muehlhausen et al. [8], the meta-analyses included the same types of electronic devices, though the way in which they were categorized differed. For example, all meta-analyses included papers that had web-based administrations. Gwaltney et al. [7] and Muehlhausen et al. [8] included these papers as part of the PC/Laptop category, while Campbell et al. [6] and the current study categorized them separately

The measurement properties of the SF-36 and SF-12 also facilitated interpretation of the observed agreement between scores. As previously noted, the meta-analysis correlations between modes of administrations ranged from 0.76 to 0.91. The test–retest reliability of the SF-36/12 domains has been shown to range from 0.66 to 0.93 for the SF-36 when using 2 weeks between administrations [41], and from 0.61 to 0.88 for the SF-36v2 using a mean time between administrations of 106 days [10]. These results indicate that the degree of agreement between modes of administration is similar, and in some cases better, than the test–retest reliability of the paper survey over both short and long intervals. The pattern of between-mode agreement and paper-based test–retest reliability was similar across domains. The role-emotional domain had the lowest agreement and reliability while physical functioning and general health domains were among the highest on both. The results of the current study, combined with previously published studies, provide substantial evidence that migrating the SF-36 and SF-12 from paper to electronic mode of administration does not substantially alter the way in which participants respond to the either the SF-36 or the SF-12 (v1 or v2).

When migrating a survey from one mode to another, the degree of modification that is required is an essential point of consideration [2], as aspects of formatting, layout, and even text size may differ between modes. Could participants’ responses be differentially affected by whether the migration was to a larger screen electronic application or device, such as a computer with full size screen, a tablet with a moderately sized screen, or a smaller handheld device like a smartphone? This concern was reported in previously published meta-analyses of PRO mode equivalence [7, 8]. The results of the current meta-analysis, however, indicate that this is not a concern for the SF-36 and SF-12 surveys, as differences between correlations were small and non-significant regardless of electronic mode used. This finding may be of particular importance to clinical trials, where the use of multiple modes of administration may occur, and for comparing findings from one study to findings from prior studies that may have used a different electronic (or paper) mode for data collection [3].

This study provides strong evidence of the measurement equivalence of the SF-36 and SF-12 across paper and electronic mode of administration. These data indicate that with a proper migration, adopting an electronic mode of the SF-36 or SF-12 should not influence participants’ responses, but researchers should carefully consider both the advantages and disadvantages of selecting one mode over another. While electronic modes confer advantages for the researcher, eliminating the need for data entry and reducing the quantity of missing data, studies that require an electronic device with internet access risk excluding individuals whose insights and experience deserve to be included in research. As such, researchers who adopt electronic modes of administration must consider the implications such a decision has on the representativeness of their study’s population, or take additional steps, such as implementing a mixed-mode design or providing the electronic device and internet access, to include participants who would be otherwise excluded.

There are a few study limitations to note. First, publication bias may reduce the number of available reports that indicate a lack of equivalence between modes, and only papers published in English could be assessed. Second, most published mode equivalence studies did not include details of exact changes made when migrating from paper to electronic format. We know from the date of publication that some studies could not have used the developer-tested single-item format electronic version, available since 2007. The study authors requested and were provided screen shots or had migration-related details for only 7 of the 25 studies. Thus, it is possible that some studies did not have a high-quality migration that maintained integrity to the original paper form. Nevertheless, the data suggest that the migrations were likely all relatively faithful to the original paper form, as one would expect far less agreement and greater mean differences if this was not the case. Third, there were not enough studies of smaller-screen devices, such as non-phone handheld devices or smartphones to investigate type of electronic mode separately, and instead tablets and handheld modes had to be combined in moderator analyses. Across studies, the specificity with which the type of electronic mode was reported was inconsistent. Fourth, there were not enough studies for any particular population to investigate if those with a particular condition or characteristic would be more vulnerable to differences in presentation of the SF-36 and SF-12 in different modes. However, the consistent findings across 25 studies, several of which were with diseased populations, or oversampled the elderly, add to the strength of our findings.

## Conclusions

Scores on the SF-36 and SF-12 show high consistency between format of administration for all scale and summary scores (PF, RP, BP, GH, VT, SF, RE, MH, MCS, and PCS), and this was found to be true regardless of intervals between administration, survey languages, and type of electronic device. The diversity of studies included in the meta-analysis is a strength of the study, and increases the generalizability of the reported results. The results of this meta-analysis provide strong evidence of the equivalence of SF-36 and SF-12 scores across paper and electronic formats.
